# Optimization of Mechanical Characteristics of Cu-35.8%Zn Brass by Rotary Swaging and Subsequent Annealing

**DOI:** 10.3390/ma19183870

**Published:** 2026-09-11

**Authors:** Natalia Martynenko, Eleonora Chistyukhina, Ivan Nikitin, Dmitry Prosvirnin, Mikhail Kaplan, Vladimir Andreev, Alexey Kolmakov, Olga Rybalchenko

**Affiliations:** 1 A.A. Baikov Institute of Metallurgy and Materials Science, Russian Academy of Sciences, 119991 Moscow, Russia; e.chistyuhina@mail.ru (E.C.); imetran@yandex.ru (D.P.); mishakaplan@yandex.ru (M.K.); vandreev@imet.ac.ru (V.A.); akolmakov@imet.ac.ru (A.K.); orybalchenko@imet.ac.ru (O.R.); 2 Laboratory of Mechanical Properties of Nanostructured Materials and Superalloys, Belgorod State University, 308015 Belgorod, Russia; nikitin_i_s@mail.ru

**Keywords:** brass, rotary swaging, annealing, microstructure, strength, ductility, fatigue limit

## Abstract

The effect of rotary swaging (RS) at room temperature and subsequent annealing at 350 °C on the microstructure, mechanical properties, and fatigue strength of Cu–35.8%Zn two-phase brass was studied. A structure with grains of α and β′ phases elongated along the deformation direction was formed after RS. It was also shown that subgrains of 200–300 nm in size, shear bands 100–200 nm wide, and deformation twins 10–30 nm wide were formed inside the α-phase grains. RS caused the increase in the yield stress (YS) from 93 ± 4 to 717 ± 6 MPa and the ultimate tensile strength (UTS) from 332 ± 2 to 744 ± 19 MPa with a decrease in ductility (El) from 71.0 ± 2.0 to 10.3 ± 1.7%. The fatigue limit also increased from 240 to 415 MPa after RS. Subsequent annealing at 350 °C induced recrystallization of the α-phase with the formation of equiaxed grains 2.2–3.6 µm in size, which resulted in a decrease in UTS to 462–466 MPa and an increase in ductility to 44–45%. Extending the annealing time to 4 h did not affect the strength and ductility values of the alloy.

## 1. Introduction

Brasses are binary or multicomponent copper-based alloys in which zinc is the main alloying element. Owing to a unique combination of properties such as high thermal and electrical conductivity, good corrosion resistance, excellent workability, and reasonable cost, brasses are widely used in various branches of modern industry (from mechanical engineering and shipbuilding to automotive, electrical engineering, construction, and consumer goods manufacturing) [1]. However, with the development of technologies that demand ever higher reliability, durability, and lightweight construction, increasingly stringent requirements are imposed on brass materials in terms of strength, ductility, and fatigue endurance. Conventional processing methods, such as casting, hot extrusion, and conventional rolling, are gradually reaching their limits, which encourages researchers to seek new routes of deformation processing that can improve the mechanical characteristics of brass without deteriorating its in-service properties.

According to their phase composition, brasses are divided into three classes: α-brass, consisting of an α solid solution of zinc in copper with an FCC (face-centered cubic) lattice; α + β-brass, which additionally contains a β solid solution based on the chemical compound CuZn with a BCC (body-centered cubic) lattice; and β-brass, composed predominantly of the β-phase [2]. The α- and β-phases differ significantly in mechanical properties and deform by different mechanisms [3]. For instance, the α-phase exhibits high ductility and is therefore readily deformable even at room temperature, whereas the β-phase is more brittle, especially at room temperature when it transforms into the ordered β′-phase [4]. Consequently, at low temperatures, deformation occurs predominantly in the more ductile α-phase, where the main mechanisms are dislocation creep, deformation twinning, and shear band formation [5,6]. However, with increasing processing temperature, in particular above about 460 °C where the β′ → β transition occurs, or during superplastic deformation, the β-phase becomes increasingly involved in the deformation process [7].

Among the many methods of deformation processing, rotary swaging (RS) has attracted increasing attention in recent years. RS is a promising processing technique that can significantly enhance the mechanical properties of metals and alloys [8]. Rotary swaging is a process of sequential reduction in a workpiece (rod, tube, or wire) using radially arranged hammers that perform high-frequency reciprocating movements, as a result of which the cross-section of the product is gradually decreased. Compared with conventional drawing or rolling, RS offers a number of unique advantages in terms of the deformation scheme. First, the workpiece is subjected to local, multiple, and multidirectional high-speed loading during rotary swaging, which promotes the activation of a greater number of slip and twinning systems and leads to more effective grain refinement. Second, RS not only allows substantial grain refinement but also enables precise control over the shape and dimensions of the finished product, making it directly suitable for industrial production. Third, RS can be easily scaled up to mass production, which gives it a significant advantage over many laboratory-scale deformation methods, for example, severe plastic deformation (SPD) techniques [9].

The advantages of using RS for improving the properties of copper and copper alloys have already been extensively investigated in a number of studies [10,11,12,13,14,15,16,17,18]. For example, it was shown that RS leads to an increase in electrical conductivity relative to the annealed state in pure copper due to the formation of a favorable texture, elongation of grains in the direction of electron motion, and the occurrence of recovery processes during deformation [10]. Moreover, it was demonstrated that grain refinement after RS allows the yield stress (YS) of pure copper to be increased to values above those of the annealed state without loss of electrical conductivity [11]. In low-alloyed electrical bronzes, RS simultaneously refines the copper matrix and promotes uniform precipitation of nanosized strengthening particles, thereby enhancing strength through grain-boundary and dispersion strengthening. For instance, in the Cu–0.5%Cr–0.08%Zr alloy (here and below, alloy compositions are given in wt. % unless otherwise stated), RS increases the ultimate tensile strength (UTS) to 597 ± 9 MPa and raises the fatigue limit to 345 MPa owing to the formation of an ultrafine-grained (UFG) microstructure and the precipitation of finely dispersed Cr and Cu_5_Zr particles [12]. In the Cu–0.5Cr alloy, RS enabled a simultaneous increase in yield stress (up to 494 MPa) and electrical conductivity (up to 82.2% IACS) due to grain refinement and accelerated chromium precipitation [13]. RS has also proved effective in other copper alloys. It was shown that as the strain degree increases during RS in the Cu–15Ni–8Sn alloy, the microstructure evolution proceeds in the following sequence: dislocations → transverse twins (ε ≥ 1.3) → lamellar fine grains (ε ≥ 1.8) → nanotwins (ε ≥ 2.2) → nanocrystals (ε ≥ 3.2) [14]. The structure after RS at ε = 3.9 is characterized by a high dislocation density and a lamellar fine-grained structure with local nanotwins and nanocrystals, and the ultimate tensile strength and yield stress increase to 1200 MPa and 1156 MPa, respectively [14]. A similar strength increase was also demonstrated for the Cu–9Ni–6Sn alloy [15]. Furthermore, it was shown using the Cu–8 wt. % Sn alloy as an example that RS leads to the formation of a lamellar ultrafine-grained α-Cu phase and the dispersion of brittle Sn-rich phases, which effectively regulates deformation and hinders crack propagation and coalescence, thereby increasing the strength of the material [16]. The promise of RS for improving the mechanical properties of brasses has also been addressed in several works. For example, it was shown that RS can increase the yield stress and ultimate tensile strength by approximately 10 and 3.5 times, respectively, while reducing ductility from 39.9 ± 3.8 to 6.3 ± 0.4% in single-phase Cu–32%Zn brass due to the formation of a UFG structure in the α-phase [17]. Subsequent half-hour annealing of the swaged alloy at 450 °C leads to a simultaneous increase in ductility to 49.1 ± 1.4% and a reduction in UTS by almost a factor of two [17]. A similar study was conducted on two-phase commercial Cu–36%Zn brass, for which it was shown that RS with a reduction in workpiece diameter from 32 to 7 mm increases its ultimate tensile strength to 820 MPa while simultaneously reducing ductility to 2.4% [18]. Subsequent heating of the alloy at 400 °C for 2 h reduces the strength to 455 MPa and increases ductility to 35% owing to textural and structural transformations [18].

The conducted studies indicate that RS is an effective method for improving the properties of copper alloys, in particular brasses. However, the specific mechanisms of the effect of RS, as well as of subsequent heating, on the relationship between the microstructure and mechanical characteristics remain insufficiently investigated. Therefore, in the present work, the effect of rotary swaging on the microstructure evolution and mechanical properties of two-phase Cu–35.8%Zn brass was studied. It is expected that the obtained results will provide a theoretical basis for optimizing the processing technology of two-phase brasses and similar α-brasses, and will also open up new prospects for further enhancement of the property combination of copper alloys.

## 2. Materials and Methods

For the purpose of the study, an α + β brass containing approximately (35.8 ± 0.14) wt. % Zn was melted. The alloy was melted using pure copper (at least 99.97 wt. %) and zinc (at least 99.975 wt. %) in an induction furnace, followed by casting of ingots (diameter 52 mm, height 200 mm) into a water-cooled cast-iron mould. Subsequently, to obtain workpieces of the required diameter, the investigated alloy was hot extruded at 630 °C to a final diameter of 20 mm. Prior to rotary swaging, the alloy was subjected to annealing at 850 °C for 2 h followed by water quenching. Quantitative analysis of the elemental composition of the alloy was carried out using a sequential wavelength-dispersive X-ray fluorescence spectrometer BRUKER S8 Tiger (Series 2) (Bruker, Karlsruhe, Germany) in vacuum according to a standard procedure with the QUANT-EXPRESS^TM^ software (Bruker, Karlsruhe, Germany). The study was carried out at three different points on the ingot and then the average value was calculated to determine the Zn concentration.

Then the quenched billets were turned on a lathe to a diameter of (19 ± 0.2) mm for deformation treatment. RS was performed on a rotary swaging machine RKM 2129.02 (UZM, Sverdlovsk, Russia)at room temperature (more details on the processing method can be found in [19]). The final diameter of the workpiece after rotary swaging was 6 mm, which corresponds to a strain ε of 2.31 (ε = ln $\frac{A_{0}}{A_{f}}$, where *A*_0_ is the initial cross-sectional area of the rod; *A_f_* is the final cross-sectional area of the rod).

The microstructure of the alloys was studied by optical and transmission electron microscopy (TEM) in the longitudinal direction of the workpiece (both after pressing with subsequent quenching and after RS). An ADF I350 optical microscope (ADF OPTICS Co., Ltd., Shenzhen, China) was used for optical microscopy studies. TEM analysis was performed on samples after RS using a JEM-2100 transmission electron microscope (Jeol, Tokyo, Japan) with an accelerating voltage of 200 kV. Foils for TEM analysis were prepared by mechanical thinning to a thickness of (0.12 ± 0.01) mm, followed by electrolytic polishing on a TenuPol 5 unit using an electrolyte containing 75% C_2_H_5_OH and 25% HNO_3_ at a temperature of –20 °C and a voltage of 10 V. Parameters of structural constituents were measured by the random secant method using Digimizer 4.5 (V6.3) (MedCalc Software, Ostend, Belgium). At least 10 fields of view were used and at least 300 grains were measured to determine the average grain size of the alloy in the initial state. At least 15 fields of view were used to evaluate the results of TEM observation.

Microhardness measurements were carried out on an automatic microhardness tester 402MVD Instron Wolpert Wilson Instruments (Wilson Instruments, Norwood, MA, USA) at a load of 100 g and an indenter dwell time of 10 s. The microhardness of the rods processed by RS was measured in the longitudinal section of the rod. The non-uniformity of deformation processing across the workpiece was examined by measuring the microhardness in steps of 1 mm from one edge of the rod to the other. Brinell hardness was measured on an IT 5010-01 testing machine (Impuls, Ivanovo, Russia) with a steel indenter of 2.5 mm diameter under a load of 62.5 kg and a dwell time of 30 s. The evaluation of hardness and microhardness parameters was carried out by measuring the parameter five times and then averaging.

Mechanical properties of the alloys were studied by uniaxial tensile testing and construction of stress–strain curves. For this purpose, flat specimens with a gauge length of (5.75 ± 0.1) mm and a cross-section of 2 × 1 mm (with an error of ± 0.1 mm), cut parallel to the deformation direction, were tested on an Instron 3382 testing machine (Instron, High Wycombe, UK). The tests were carried out at room temperature using at least three specimens per condition.

Fatigue strength tests were performed under repeated tension loading on an Instron Electropuls E3000 testing machine (Instron, High Wycombe, UK)equipped with WaveMatrix™3 (Instron, High Wycombe, UK), using flat specimens with a working cross-section of 1 × 1 mm (with an error of ±0.1 mm) and a gauge length of (4.46 ± 0.1) mm. The tests were conducted up to 10^7^ loading cycles at a frequency of 30 Hz (sinusoidal waveform) and a stress ratio of R = 0.1. The fatigue limit of the alloy was defined as the stress at which at least four specimens withstood 10^7^ loading cycles without fracture.

## 3. Results

**Figure 1:**
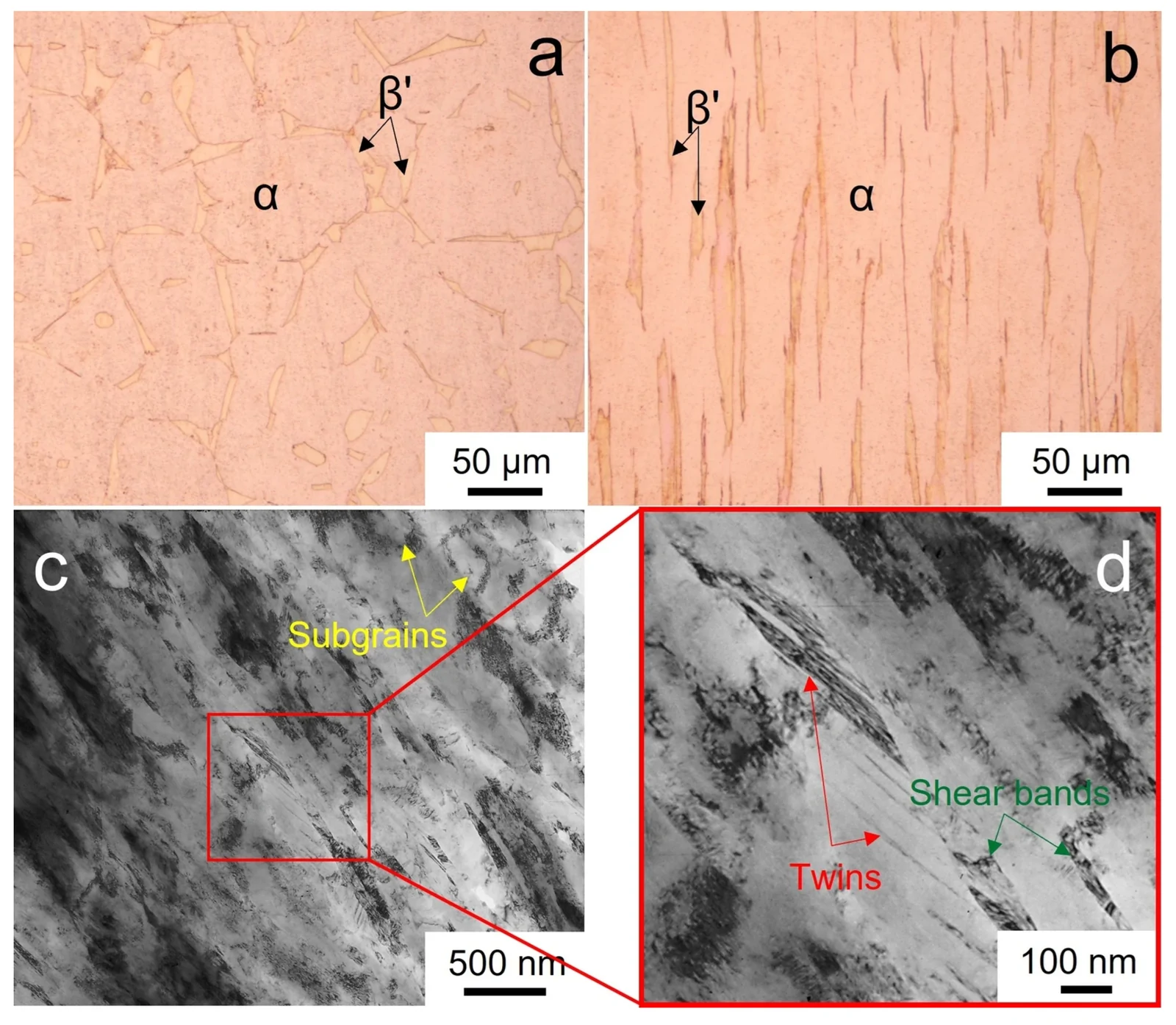
Microstructureof Cu-35.8%Zn alloy after quenching (**a**) and subsequent rotary swaging (**b**–**d**).

Microstructure investigation of the Cu–35.8%Zn alloy before and after deformation by RS method at room temperature presents in Figure 1.

The alloy has a two-phase structure consisting of irregularly shaped α-phase grains surrounded by a thin interlayer of β′-phase in the initial as-quenched state. The average size of the α-phase grains in this case is 41 ± 5 μm, and the width of the β′-phase is 12 ± 1 μm with a volume fraction of ~15.3% (Figure 1a). The structure of the alloy elongates along the deformation direction during RS, forming long elongated grains several hundred micrometers in length. The average width of the α-phase grains is 23 ± 2 μm, while that of the β′-phase is 4 ± 1 μm. In addition, a slight decrease in the volume fraction of the β′-phase to about 11.4% is observed (Figure 1b). Further microstructure studies using TEM analysis revealed the formation of an ultrafine-grained (UFG) structure within the elongated α-phase grains (Figure 1c,d). This UFG structure consists of zones with fine subgrains about 200–300 nm in size, shear bands 100–200 nm wide and up to several micrometers long, as well as deformation twins from 10 to 30 nm (Figure 1d).

**Figure 2:**
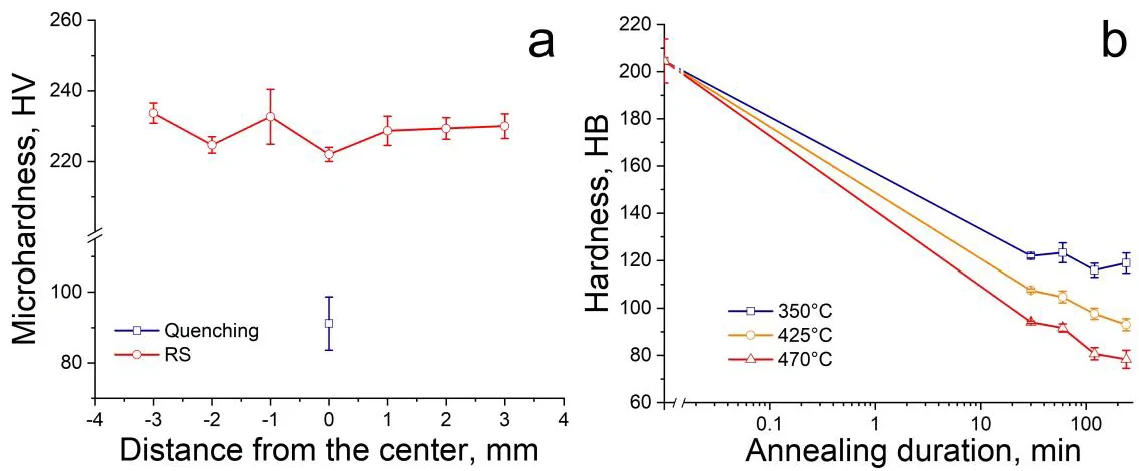
Microhardnessof the alloy before and after RS (**a**), as well as the dependence of the Brinell hardness of the alloy after RS on the heating duration at 350, 425, and 470 °C (**b**).

To assess the degree of workability of the workpiece, a study of the alloy microhardness after RS was conducted depending on the distance from the center of the workpiece (Figure 2a). The obtained results showed that the microhardness of the alloy after RS has fairly close values and differs only slightly at the center of the rod and at its periphery. The minimum value measured at the center of the workpiece was 222 ± 2 HV, while the maximum was 234 ± 3 HV, which corresponded to the edge of the workpiece. Moreover, RS significantly increases the microhardness relative to the quenched state: from 91 ± 8 HV to 229 ± 4 HV (average value over the cross-section of the workpiece).

Figure 2b presents the results of the study of the dependence of the hardness of the deformed alloy on the heating duration at 350, 425, and 470 °C. The investigations showed that heating at all selected temperatures for just half an hour already leads to a substantial decrease in the hardness of the alloy. Moreover, the higher the heating temperature, the more intense the reduction in hardness, as can be judged from the slope of the curves in Figure 2b. Thus, the hardness of the alloy was 205 ± 9 HB after RS at room temperature. Heating for 30 min results in a drop in hardness to 122 ± 1 HB, 108 ± 1 HB, and 94 ± 0.5 HB for 350, 425, and 470 °C, respectively. With a further increase in heating duration to 4 h, at temperatures of 425 and 470 °C a slight decrease in hardness is observed, to 93 ± 3 HB and 78 ± 4 HB, respectively, whereas for heating at 350 °C, the hardness values after 4 h of heating do not change compared with the annealing for 30 min. The hardness of the deformed alloy after heating at 350 °C for 4 h was 119 ± 4 HB (Figure 2b).

**Figure 3:**
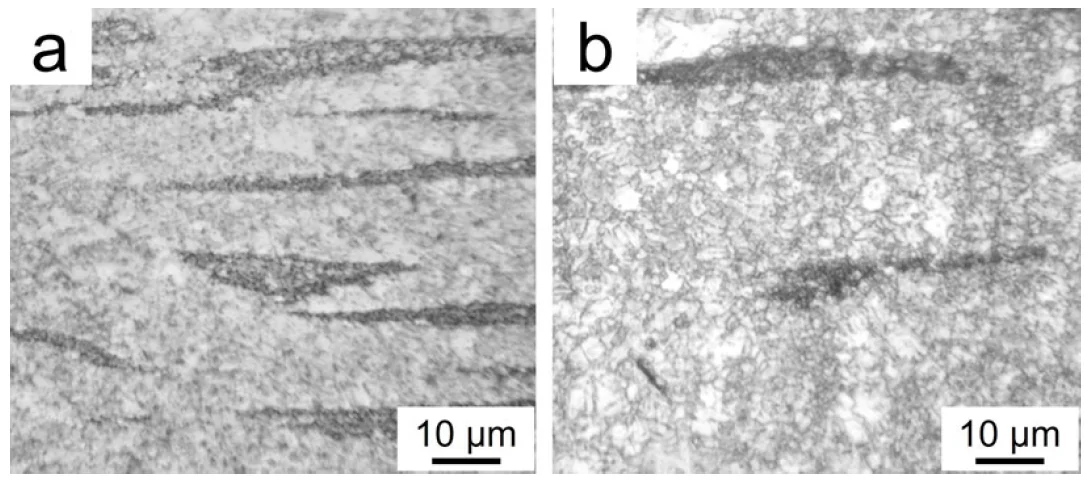
Optical microscopy of the alloy after RS and annealing at 350 °C for 30 min (**a**) and 4 h (**b**).

Figure 3 shows the microstructure of the Cu–35.8%Zn alloy after RS and additional annealing at 350 °C for 30 min and 4 h. Optical microscopy studies showed that the structure of the alloy after RS and additional annealing at 350 °C also consists of α- and β′-phases elongated along the deformation direction. Within the elongated α-phase, predominantly equiaxed grains with a size of 2.2 ± 0.3 μm are formed after annealing for 30 min, and 3.6 ± 0.1 μm after annealing for 4 h. It should also be noted that the annealing duration does not significantly affect the width of the β′-phase, which is 3–5 μm for both considered conditions.

**Figure 4:**
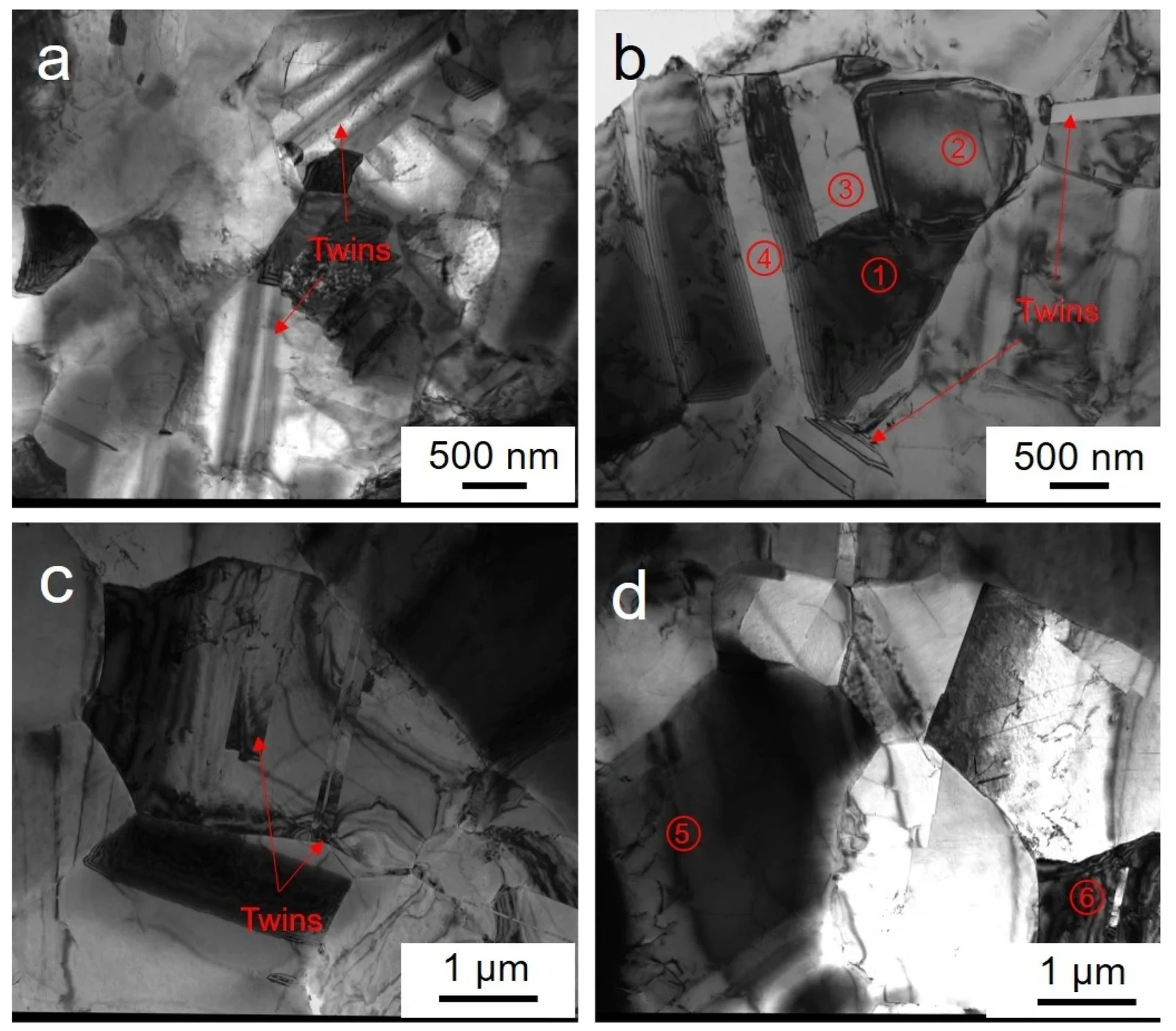
TEMimages of the alloy structure after RS and annealing at 350 °C for 30 min (**a**,**b**) and 4 h (**c**,**d**). The red numbers indicate the points at which TEM-EDS analysis was performed (see Table 1).

The results of the microstructure study by TEM analysis also confirm the occurrence of recrystallization processes in the alloy after RS and additional heating at 350 °C (Figure 4).

The formation of individual recrystallized grains ranging in size from 500 nm to 2.5 μm, as well as the formation of twins from 100 nm to 500 nm wide is observed in the structure after RS and heating for 30 min (Figure 4a,b). Increasing the heating duration to 4 h leads to more complete recrystallization (Figure 4c,d). In this case, the structure is largely recrystallized, and the grain size is about 1–3.5 μm. Additionally, deformation and annealing twins are also present in the structure. The results of TEM-EDS analysis confirm that recrystallization occurs within the α-phase grains (Table 1). It should be noted that the β′-phase inclusions had a width of 3–5 μm and a length ranging from 20 to several hundred microns, both after rotary swaging and after subsequent annealing, and did not undergo fragmentation. Owing to this relatively large size, they were not the subject of TEM investigations. Moreover, according to the Cu–Zn phase diagram, only the α- and β′-phases are expected in this alloy, the presence of other phases is highly unlikely, and thus the identification of the β′-phase does not require additional TEM-EDS confirmation.

**Figure 5:**
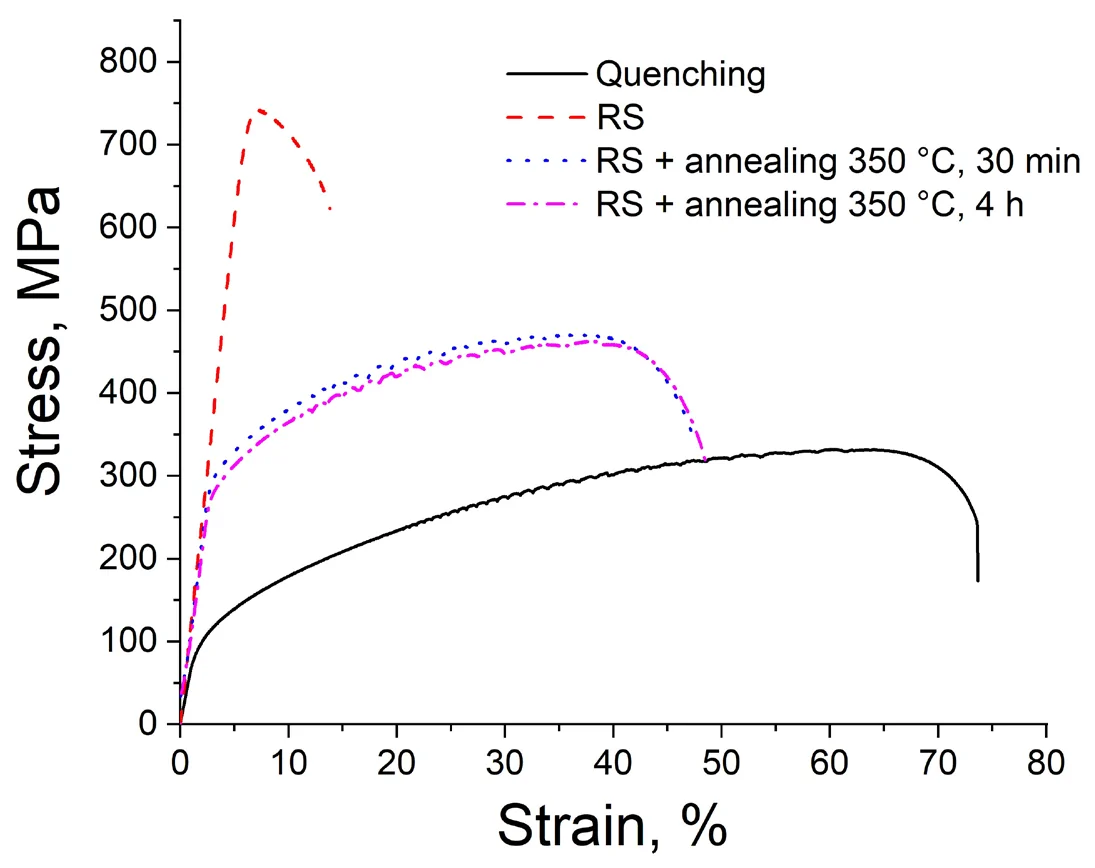
“Stress–strain” curves of the alloy in different states.

Table 2 and Figure 5 present the results of the investigation of the mechanical properties of the alloy after quenching, RS, and subsequent annealing at 350 °C for 30 min and 4 h. The yield stress of the alloy is 93 ± 4 MPa, and the ultimate tensile strength is 332 ± 2 MPa with an elongation of 71 ± 2% in the as-quenched state. After RS at room temperature, the yield stress and ultimate tensile strength increased to 717 ± 6 MPa and 744 ± 19 MPa, respectively. However, this increase in strength was accompanied by a substantial decrease in elongation (to 10.3 ± 1.7%). Subsequent annealing leads to a reduction in YS by more than half and in UTS by 35–40%. Simultaneously, a significant increase in ductility is observed, which increases almost fourfold, reaching ~45%. It should be noted that the mechanical properties of the alloy after annealing for 30 min and 4 h show only minor differences and remain the same within the error limits.

**Figure 6:**
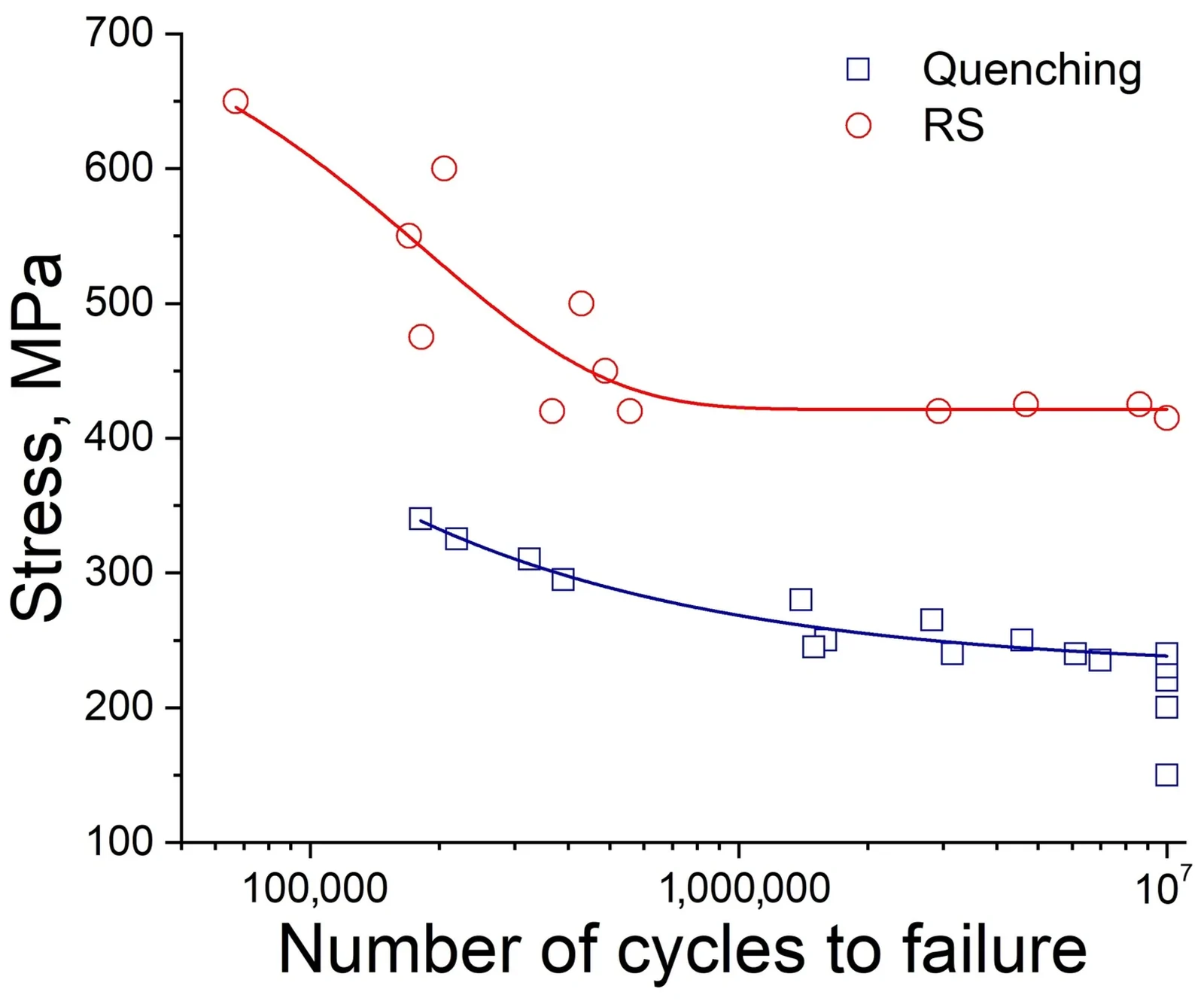
Fatiguestrength of the alloy before and after RS.

Figure 6 shows the results of the study of the fatigue strength of the alloy in the quenched state and after RS.

The conducted studies showed that RS of the alloy leads to a significant increase in its fatigue strength. In the as-quenched state, the fatigue limit of the alloy is 240 MPa. An increase in the fatigue limit to 415 MPa is observed after rotary swaging.

## 4. Discussion

A systematic study of the effect of cold rotary swaging (room temperature) and subsequent annealing at 350 °C on the microstructure, mechanical properties, and fatigue strength of two-phase Cu–35.8%Zn brass was conducted in this work. The Cu–35.8%Zn alloy exhibits a structure typical of two-phase brasses, consisting of relatively coarse α-phase grains separated by layers of β′-phase in the as-quenched state. After RS, the α- and β′-phase grains become elongated along the deformation direction, and within the α-phase an ultrafine-grained structure, consisting of fine subgrains, shear bands, and nanotwins is formed. This fact is probably related to the difference in plastic properties of the α- and β′-phases at room temperature. The α-phase (FCC lattice) has a high deformability. Therefore, large strains accumulate inside the grains in this case, leading to substantial refinement. On the other hand, the β′-phase (ordered BCC structure) is relatively brittle and, upon cold deformation, partially fractures, dissolves, or even undergoes phase transformations. In our case, the volume fraction of the β′-phase slightly decreases (to 11.4%) after RS, probably due to partial local dissolution. While traditional phase transformations are largely dependent on thermal diffusion, plastic deformation accelerates or modifies this balance through strong lattice distortion. Similar behavior has been previously reported in copper alloys, for example in [20,21,22], where possible dissolution of intermetallic phase or its transition to a non-equilibrium state under high stresses was indicated. The most interesting result is the formation within the elongated α-grains of an ultrafine-grained structure consisting of subgrains 200–300 nm in size, shear bands 100–200 nm wide, and deformation twins 10–30 nm wide. Such a multilevel hierarchical microstructure is characteristic of severe plastic deformation of metals with low stacking-fault energy, to which α-brass belongs. A complex stress–strain state with a high strain rate and frequent stress reversal is realized under RS conditions, which activates multiple slip and twinning systems. The formation of nano-thick twins is particularly important, since they serve as effective barriers to dislocation motion and simultaneously contribute to additional grain refinement. Similar observations were made in [23,24], where substantial twinning was also observed after deformation. At the same time, the predominant formation of the UFG structure in the α-phase is explained by the fact that, unlike the β′-phase, a larger number of high-angle boundaries accumulate in it during deformation due to easier dislocation accumulation [25].

The formation of such a structure provides a significant increase in static strength (YS increases from 93 ± 4 to 717 ± 6 MPa (by a factor of 7.7), UTS—from 332 ± 2 to 744 ± 19 MPa (by a factor of 2.2), microhardness—from 894 ± 74 to 2244 ± 41 MPa) and fatigue endurance (from 240 to 415 MPa), but substantially reduces the ductility of the alloy (from 71.0 ± 2.0% to 10.3 ± 1.7%). At the same time, subsequent annealing restores ductility while maintaining a sufficiently high level of strength. Such a significant strengthening cannot be explained by any single mechanism and is the result of a superposition of several simultaneously acting factors, among which:

- Grain boundary strengthening (Hall–Petch effect). The refinement of the structure provides the main contribution to the increase in yield stress. The formation of subgrains 200–300 nm in size in the α phase may significantly increase the strength. According to estimates, if the Hall–Petch coefficient for two phase brass is taken as ~14 MPa × mm^1/2^ [26], then reducing the grain size from 41 μm to ~0.25 μm (the average subgrain size) can increase the yield stress by ~800 MPa. However, it should be taken into account that the structure of the alloy after RS is not homogeneous and does not consist entirely of subgrains, but also shear bands and slightly deformed areas. Therefore, the calculated value is only an estimate and greatly exaggerated.
- Strain hardening (dislocation hardening). The high dislocation density accumulated during RS significantly increases the flow stress. The absence of intermediate heating of the workpiece between RS passes helps to preserve the high accumulated dislocation density, which additionally increases the strength. At the same time, regions with high local strain are observed inside the subgrains, indicating the absence or incompleteness of dynamic recovery at room temperature.
- Twinning. The formation of nanotwins 10–30 nm wide after RS contributes through the twinning mechanism, which for brasses is one of the main mechanisms at low temperatures. Twin boundaries, like high angle boundaries, act as obstacles to dislocation slip, and their high density leads to additional strengthening.
- Dispersion strengthening. Although there are no explicitly precipitated particles in the alloy, the redistribution of alloying elements between the α and β′ phases, as well as possible enrichment of grain boundaries with zinc, can create additional barriers to dislocation motion.

It should be noted that such strong strengthening is accompanied by a sharp drop in ductility (by almost a factor of 7), which is typical for nanostructured and ultrafine-grained materials. The limited strain-strengthening capacity in the UFG state leads to early strain localization and a reduction in uniform elongation during tensile testing. Nevertheless, the achieved ductility value (~10%) for such a high-strength state is quite acceptable [27]. In the work of Al-Hamdany et al. [18], a strength of up to 820 MPa with a ductility of 2.4% was achieved on commercial two-phase Cu–36%Zn brass after RS from 32 to 7 mm (ε = 3.04, which is higher than in our case). Our approach, with an intermediate degree of deformation and subsequent mild annealing, is more balanced and provides high ductility, making the material suitable for products operating under dynamic loads.

It is also important to note that the fatigue limit (at 10^7^ cycles, R = 0.1) increases from 240 MPa to 415 MPa, i.e., by 73% after RS. The improvement in fatigue characteristics after RS is primarily associated with grain refinement: a large number of grain and subgrain boundaries hinder the initiation and propagation of fatigue cracks. In addition, compressive residual stresses arise on the surface of the samples during RS, which are known to have a positive effect on the fatigue resistance of metals and alloys [28,29]. The formation of nanotwins also plays an important role, as they serve as effective barriers to dislocation motion under cyclic loading, slowing down damage accumulation. In two-phase (α + β) brasses, a fatigue crack generally nucleates in the softer α-phase, which has lower strength and deforms more readily under cyclic loading [30,31]. It was shown that plastic slip actively develops along {111} planes in the α-phase, whereas in the β-phase, slip is significantly less pronounced and becomes more active only after a large number of cycles [30,31]. Kawazoe et al. showed that the predominant slip in the α-phase occurred on the primary (111) plane, while in the β-phase it tended to occur on planes other than the ($\bar{1}$01) plane, namely quite close to the ($\bar{2}$11) plane [30]. It was also shown that the main crack initiation sites are either the α-phase itself, where the accumulation of dislocation damage is the most intense, or the α/β interphase boundaries, which are zones of stress concentration and structural heterogeneity [32]. Given that the RS process resulted in substantial hardening primarily in the soft α-phase due to the formation of an ultrafine-grained (UFG) structure, nanotwins, and shear bands, an increase in fatigue strength is to be expected.

Subsequent annealing at 350 °C for 30 min and 4 h leads to a radical change in the structure and properties. The yield stress of the alloy decreases to 278–292 MPa, and the ultimate tensile strength to 462–466 MPa. At the same time, however, a substantial increase in ductility (to 44–45%) is observed. These changes are due to the development of recrystallisation processes in the α-phase. Structural analysis showed that during annealing for 30 min, equiaxed recrystallized grains about 2 μm in size are formed within the elongated α-grains, and their size grows to ~3 μm with an increase in time to 4 h. It is important to emphasize that no significant change in mechanical properties is observed with increasing annealing duration. This may indicate that after 30 min at 350 °C, recrystallisation has already partially taken place, and further grain growth proceeds rather slowly. Such behavior is explained by the fact that a large degree of strain is accumulated in the α-phase after RS, and the numerous dislocations, subgrain boundaries, and twins serve as effective nucleation sites for new grains. Therefore, recrystallisation proceeds at a high rate even at a relatively low temperature.

Interestingly, the width of the β′-phase after annealing remains practically unchanged (3–5 μm), and its volume fraction does not recover to the initial 15.3%. This indicates that the β′-phase does not undergo a reverse phase transformation or noticeable growth at 350 °C, retaining its elongated morphology. This behavior is explained by the fact that diffusional redistribution of zinc and copper in the β′-phase requires higher temperatures or longer holding times. Thus, the structure is a mixture of recrystallized α-phase with equiaxed grains (2–3 μm in size) and undeformed residual β′-layers after annealing. The presence of hard β′-layers, which have retained their elongated shape, may explain why the yield stress (~280 MPa) and ultimate tensile strength (~460 MPa) remain noticeably higher after annealing than in the initial as-quenched state (93 MPa and 332 MPa). In addition, the recrystallized α-grains have a size of ~2–3 μm, which is significantly smaller than the initial 41 μm, meaning that a certain contribution from grain-boundary strengthening is retained. Additional strength is also provided by the presence of annealing and deformation twins. The combination of these factors ensures a unique balance of high ductility (44–45%) with sufficiently high strength.

The obtained results are in good agreement with recent studies on the application of rotary swaging to copper alloys. For single-phase Cu–32%Zn brass [17], it was shown that RS increases the yield stress by approximately 10 times and the ultimate tensile strength by 3.5 times, with a reduction in ductility to ~6%, while subsequent annealing at 450 °C restores ductility to 49% with a twofold reduction in strength. In our case, for two-phase Cu–35.8%Zn brass, RS gives an increase in yield stress by a factor of 7.7 and in ultimate tensile strength by a factor of 2.2, with a final ductility of 10.3%, while annealing at a lower temperature (350 °C) allows a higher strength (~460 MPa) to be retained at a similar ductility (~45%). This advantage is apparently associated with the presence of the β′-phase, which does not recrystallize at 350 °C and serves as an additional strengthener, as well as with the more moderate annealing temperature, which limits grain growth. Results close to ours were also obtained on ECAP-processed Cu–40%Zn brass additionally annealed at 350 °C for 20 min (UTS ~500 MPa, YS ~304 MPa, and El ~42%) [33]. However, from the standpoint of practical application, the combination of RS and annealing at 350 °C represents a technologically simple and scalable method for producing high-strength and simultaneously ductile brass.

Thus, the conducted study not only reveals the main regularities of structure and property formation in two-phase brass during rotary swaging and subsequent annealing, but also lays the foundation for the development of optimized processing regimes that ensure the required combination of service characteristics.

## 5. Conclusions

1. Rotary swaging at room temperature (ε = 2.31) of two-phase Cu–35.8%Zn brass leads to the formation of a structure elongated along the deformation direction, with the width of α-phase grains decreasing from 41 ± 5 μm to 23 ± 2 μm, and the β′-phase from 12 ± 1 μm to 4 ± 1 μm. At the same time, the volume fraction of the latter decreases from 15.3 to 11.4%.
2. An ultrafine-grained structure consisting of subgrains 200–300 nm in size, shear bands 100–200 nm wide, and deformation twins 10–30 nm in width is formed inside the elongated α-phase grains.
3. RS leads to significant strengthening of the alloy. Microhardness increases from 894 ± 74 to 2244 ± 41 MPa, yield stress increases from 93 ± 4 to 717 ± 6 MPa, and ultimate tensile strength increases from 332 ± 2 to 744 ± 19 MPa. At the same time, ductility naturally decreases from 71.0 ± 2.0 to 10.3 ± 1.7%.
4. The fatigue limit (10^7^ cycles, R = 0.1) of the alloy after RS increases from 240 to 415 MPa.
5. Subsequent annealing after RS at 350 °C induces recrystallization in the α-phase with the formation of equiaxed grains of 2.2 ± 0.3 μm (after 30 min) and 3.6 ± 0.1 μm (after 4 h). This leads to a decrease in strength characteristics (YS to ~280–290 MPa, UTS to ~460–470 MPa) and an increase in ductility to ~44–45%. Increasing the annealing duration from 30 min to 4 h does not result in a significant change in mechanical properties.
6. The proposed regime of “rotary swaging + annealing at 350 °C” enables the production of two-phase brass with a unique combination of high ductility (~45%) and enhanced strength (~460 MPa), making it promising for manufacturing critical components in automotive, plumbing, and electrical engineering applications that operate under dynamic and cyclic loading, while the technology is readily scalable for industrial use.

## Figures and Tables

**Table 1:** Results of TEM-EDS analysis of the alloy after RS and subsequent annealing at 350 °C.

Spectrum	Cu, wt. %	Zn, wt. %	Total, wt. %
Spectrum 1	66.41	33.59	100.00
Spectrum 2	66.16	33.84	100.00
Spectrum 3	65.78	34.22	100.00
Spectrum 4	65.94	34.06	100.00
Spectrum 5	65.27	34.73	100.00
Spectrum 6	66.06	33.94	100.00

**Table 2:** Mechanical properties of the alloy in various conditions.

State	YS, MPa	UTS, MPa	El, %
Quenching	93 ± 4	332 ± 2	71.0 ± 2.0
RS	717 ± 6	744 ± 19	10.3 ± 1.7
RS + annealing 350 °C, 30 min	292 ± 5	466 ± 7	44.3 ± 0.6
RS + annealing 350 °C, 4 h	278 ± 5	462 ± 1	45.0 ± 0.1

## Data Availability

The original contributions presented in this study are included in the article. Further inquiries can be directed to the corresponding author.
